# Editorial: Reviews in psychiatry 2023: schizophrenia

**DOI:** 10.3389/fpsyt.2024.1444818

**Published:** 2024-07-04

**Authors:** Massimo Tusconi, Dennis Kätzel, Teresa Sánchez-Gutiérrez

**Affiliations:** ^1^ University Hospital of Cagliari, Cagliari, Italy; ^2^ Institute of Applied Physiology, Faculty of Medicine, University of Ulm, Ulm, Germany; ^3^ Department of Psychology, University of Córdoba, Córdoba, Spain

**Keywords:** schizophrenia, biomarkers, therapeutic targets, systematic reviews, psychosocial functioning, psychological therapy, symptom domains, pharmacological therapy

Schizophrenia is a severe psychiatric disorder that imposes a considerable burden on patients, their families, and society as a whole (1). The fact that the burden of schizophrenia is compounded by the insufficient efficacy of available treatment options has motivated a wide range of clinical and pre-clinical research endeavors. The contributions to this Research Topic represent this diversity of research and thereby mirror the complexity of the disorder itself. Schizophrenia is a nosological entity with a very heterogeneous spectrum of deficits. These are categorically identified as positive, negative, and cognitive symptoms (2). Moreover, recent discoveries regarding inherent biomarkers (3) and neurodevelopmental disease progression (4) increasingly suggest possible overlap with other psychiatric disorders, such as bipolar disorder (5–7). This overlap ranges from genetic (5) and environmental (6, 7) risk factors to pathophysiological mechanisms and symptoms (8, 9). Recognition of symptomatologic patterns and familiarities between disorders may provide a more precise diagnostic framing and better management of clinical aspects. It is important to identify factors that can support the differential diagnosis and allow the most appropriate treatment for Schizophrenia patients who represent nosographic clusters of considerable complexity (10). Thus, an overview of both quantitative and qualitative data on clinical characteristics and treatment outcomes may elucidate the patterns and mechanisms of Schizophrenia pathogenesis and its modifiability (11, 12). In this regard, the reviews included in this Research Topic allow for an in-depth reflection on several aspects of mental pathology in general and highlight possible future directions of scientific advancement (13).

Several reviews have examined the effectiveness of non-pharmacological treatment options for Schizophrenia. For example, in parallel with standard psychosocial and pharmacological treatment, the use of virtual reality offers new solutions that can lead to appreciable results in various functional and symptomatic domains. The review by Holopainen et al. on the efficacy of immersive extended reality (XR) interventions on different symptom domains of Schizophrenia spectrum disorders showed that treatment gamification allows for greater patient engagement in therapy, harnessing the motivation of novelty represented by this virtuous technology. Furthermore, unlike drug treatment which is often associated with many side effects and stigma, this therapeutic approach is believed to be devoid of such drawbacks and likely to provide a favorable end-user experience and greater overall adherence to the treatment. Similarly, Cao and Zhou described the effects of computerized cognitive remediation therapy (CCRT) on mental time travel in patients with Schizophrenia. Their review highlighted how this treatment may prove to be a relatively simple, inexpensive and effective way to improve symptoms.

In contrast, Tyssedalu et al. highlighted how interventions with dogs for adults diagnosed with Schizophrenia can improve their quality of life, well-being, and several positive and negative symptoms, including features associated with the severity of psychosis. However, the results of some of the reviewed studies should be interpreted with caution.

In a systematic review conducted on the treatment effects of adjunctive group music therapy in inpatients with chronic Schizophrenia, Lam et al. found that, as an add-on to standard treatment, this intervention can produce an additional improvement. Several of the 13 randomized controlled trials included in the review found beneficial effects at the level of positive symptoms - particularly auditory hallucinations -, cognitive function – especially attention - and/or negative symptoms. For the latter, improvements in avolition, social withdrawal, anhedonia, and self-care were particularly evident. At the level of subjective perception, patients also reported improvements in energy, mood, anxiety, relaxation, and quality of life.

Exploring the possibility of alternative pharmacological treatments, Bortoletto et al. reviewed the evidence for the role of the endogenous lipid palmitoylethanolamide (PEA) in psychosis. Although it does not activate the cannabinoid receptors CB1 and CB2 directly, it shares several other targets with classical endocannabinoids and enhances the availability of the latter (*entourage effect*). As it has been suggested that disruption of the endocannabinoid (eCB) system may be implicated in the etiopathogenesis of psychosis, PEA may represent a better-tolerated antipsychotic agent acting through the eCB system. In addition to its well-known analgesic properties, PEA also exerts neuroprotective and anti-inflammatory effects that may ameliorate the pathological development of Schizophrenia. Evidence suggests that PEA may specifically improve manic and negative symptoms, including apathy. Importantly, no serious adverse effects were reported in any of the human studies reviewed, suggesting a very beneficial safety profile for PEA.

Several other reviews have investigated the symptomatology of schizophrenia and related disorders. Motut et al. conducted a meta-analysis that revealed a significant correlation between social cognition and metacognition in subjects with Schizophrenia Spectrum Disorder. They were able to identify this association in various cognitive and social domains particularly those related to theory of mind, attribution and emotion processing, while no correlations emerged with indicators of cognitive intuition, self-reflexivity, or understanding others’ minds. Beyond symptomatology, the authors also noted that metacognitive training and insight therapy represent non-pharmacological interventions that may benefit social cognition and possibly other cognitive functions in individuals with Schizophrenia Spectrum Disorders.

In addition, Di Luzio et al. reviewed the clinical features and comorbidities of *very early onset Schizophrenia* (VEOS) and reported that it appears to be very similar to *early-onset* (EOS) and *adult-onset* forms of Schizophrenia (AOS). However, VEOS has some peculiar characteristics, especially a greater presence of visual hallucinations and more common resistance to conventional treatment in female patients. Moreover, men and women are equally likely to develop VEOS, which differs from the usual 1.5 times higher prevalence of general Schizophrenia in men. Guiral et al., in turn, emphasized the critical role of neuropsychological dimensions related to alterations in verbal self-monitoring of language production in Schizophrenia patients. A general consensus emerged from the review that language processing and associated mechanisms of verbal self-monitoring are not deemed secondary, but rather fundamental to the disorder. A particularly clear link with emotional and cognitive dimensions, such as perception, is reported, and accompanying neurophysiological measurements have revealed the involvement of frontotemporal networks and regions such as the insula, amygdala, putamen and cingulate cortex. Based on these findings the authors suggested the development of neuropsychological techniques and tests for a better diagnosis and treatment of Schizophrenia.

Similarly, Calciu et al. investigated the psychotic phenomenon of dissociation and its relationship to recovery from psychosis. The authors reported that dissociative psychotic experiences are a very complex phenomenon that involves multiple mechanisms and influences recovery, whereas this field appears to be clearly understudied.

Finally, Hui et al. reviewed studies comparing Delusional Disorder (DD) and Schizophrenia and reported that, overall, no differences emerge between age-matched and non-age-matched features. However, compared to Schizophrenia, DD is associated with generally better outcomes in terms of psychopathology and functioning.

In summary, the reviews included in this Research Topic highlight a number of aspects and draw synoptic conclusions that are not easily identifiable in individual research articles given the considerable methodological and conceptual diversity in Schizophrenia research. For example, more confidence could be gained regarding the effectiveness of alternative or adjunct treatment approaches, such as VR- or animal-based therapy, computerized cognitive remediation therapy, or music therapy. Furthermore, the summaries of current evidence provided for rather understudied conditions in the Schizophrenia-related spectrum such as VEOS, dissociation, verbal self-monitoring, metacognition, and Delusional Disorder point to future research needs and directions. Overall, the reviews underscore that an approach to complex mental pathologies like Schizophrenia or the psychotic spectrum (14) based solely on isolated symptomatic aspects appears to be suboptimal. Therapies using multiple technologies and integrated approaches promise that treatment can be more comprehensive and humanely respectful. It is also evident that the integration of multiple aspects can stimulate the scientific community to develop new strands of clinical, basic and technological research with a view to achieving an increasingly optimal outcome in terms of satisfactory symptom management and improved quality of life.

## Author contributions

MT: Conceptualization, Formal analysis, Methodology, Writing – original draft, Writing – review & editing. DK: Writing – review & editing. TS-G: Writing – review & editing.
